# Supplementary material for: Dissecting the roles of calcium cycling and its coupling with voltage in the genesis of early afterdepolarizations in cardiac myocyte models
Source: PLoS Comput Biol. 2024 Feb 28;20(2):e1011930. doi: 10.1371/journal.pcbi.1011930 (PMC10927084; doi:10.1371/journal.pcbi.1011930)
Supplement: S1 Text — The supplemental information contains the following information: i) Simulation results of bidirectional regulations of Vm and submembrane Ca in different models; ii) the types of EADs; and iii) Control parameters and initial values of each model. (PDF) [file pcbi.1011930.s001.pdf]

Supplemental Information

The supplemental material contains the following information: i) Simulation results of bidirectional regulations of voltage (V_m) and submembrane calcium (Ca) in different models; ii) the types of EADs in different models; and iii) Control parameters and initial values of each model.

Bidirectional regulations of V_m and Ca

V_m and Ca regulate each other via the associated currents, mainly the L-type Ca current ($I_{Ca,L}$) and the sodium-calcium-exchange current (I_{NCX}). In order to reveal their effects, we clamp one of the two at different levels separately to assess how one affects the other. The default parameter sets are given in the parameter section below.

1) *Ca-$I_{Ca,L}$-Vm regulation.* Increase of Ca can suppress $I_{Ca,L}$ via Ca-dependent inactivation gate f_{Ca} in all the models, as shown in Fig A. In turn, V_m can affect Ca via modulating $I_{Ca,L}$. In the LRd, H_{UCLA}, TP04 and ORd models, when V_m is above -20 mV, $I_{Ca,L}$ quickly decreases to zero [Figs B(a)-B(d)], whereas in the GB and WG models, $I_{Ca,L}$ does not decrease to zero and even increases within a certain range of V_m [Figs B(e) and B(f)]. This is caused by the difference in the steady-state inactivation curve of the Ca channel (i.e., f_{ss}) in the AP models. In the LRd, H_{UCLA}, TP04 and ORd models, they are sigmoidal functions [see the inset of Fig B(b)], whereas in the GB and WG models, they are non-monotonic and increase with V_m when V_m is high [see the inset of Fig B(e)]. Since $I_{Ca,L}$ controls Ca entry into the submembrane space, the effect of V_m on Ca via $I_{Ca,L}$ is different in the two types of models, as shown in Fig C. We can see that in the LRd, H_{UCLA}, TP04 and ORd models, V_m exhibits a negative effect on Ca for V_m>-20 mV (i.e., larger V_m reduces $[Ca]_{sub}$), whereas in the GB and WG models, the effect is positive within a certain range (the GB model can even exhibit Ca-oscillation). This makes the GB and WG models difference from the other four models in V_m and Ca coupling and thus the Ca cycling dynamics.

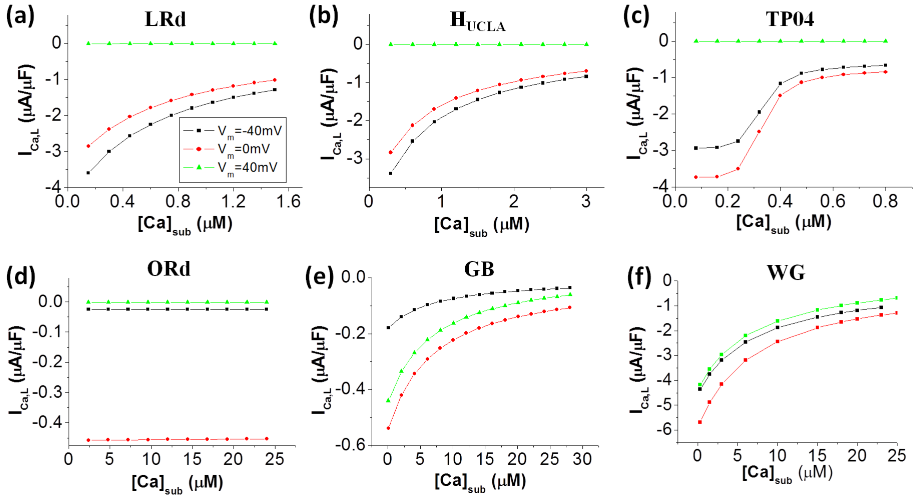

Fig A: Effects of Ca on peak $I_{Ca,L}$. V_m is clamped at -40 mV, 0 mV, and 40 mV. Under any clamped V_m, $[Ca]_{sub}$ suppresses $I_{Ca,L}$. This still holds in ORd model even though the effect is very small.

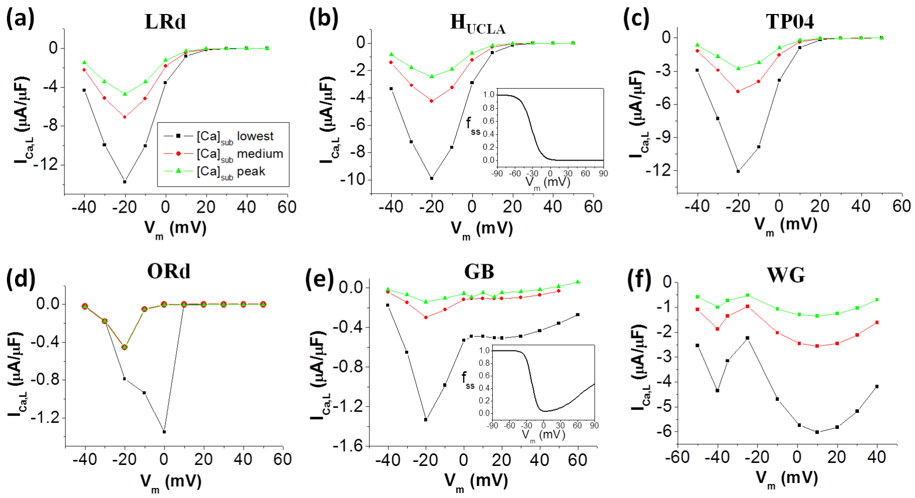

Fig B: Effects of V_m on $I_{Ca,L}$. The different clamped $[Ca]_{sub}$ values are chosen as the lowest, medium, and peak values of the Ca transient. The clamped $[Ca]_{sub}$ values are: (a) 0.02 μM, 0.6 μM, and 1.2 μM (LRd); (b) 0.3 μM, 1.5 μM, and 3 μM (H_{UCLA}); (c) 0.08 μM, 0.4 μM, and 0.8 μM (TP04); (d) 0.2 μM, 1.2 μM, and 2.4 μM (ORd); (e) 0.2 μM, 25 μM, and 60 μM (GB); (f) 0.3 μM, 10 μM, and 25 μM (WG). The insets of panels (b) and (e) are f_{ss} of the H_{UCLA} and GB models, respectively, representing two types of the models of the f_{ss} curve.

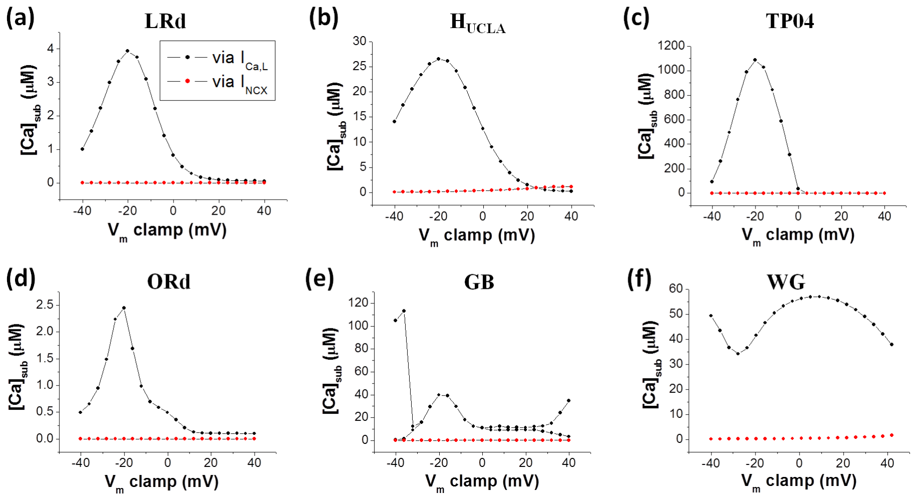

Fig C: Effects of V_m on $[Ca]_{sub}$ through $I_{Ca,L}$ (black) or I_{NCX} (red) exclusively [i.e., except $I_{Ca,L}$ (or I_{NCX}) the other currents coupled with $[Ca]_{sub}$ are set as 0]. In the GB and WG models, the effect of V_m on Ca is non-monotonic.

2) *Ca-I_{NCX}-V_m regulation*. Increase of Ca increases the amplitude of I_{NCX}, as shown in Fig D. Since I_{NCX} is an inward current which tends to depolarize V_m, then Ca→I_{NCX}→V_m forms a positive regulatory way. On the other hand, V_m's effect on $[Ca]_{sub}$ via I_{NCX} is weak (see the red dots in Fig C for comparison with that of $I_{Ca,L}$). It indicates that the V_m-I_{NCX}-Ca regulation is much weaker than that of V_m-$I_{Ca,L}$-Ca. Therefore, we can just ignore the feedback of Ca-I_{NCX}-V_m-Ca in the analysis of type III EAD.

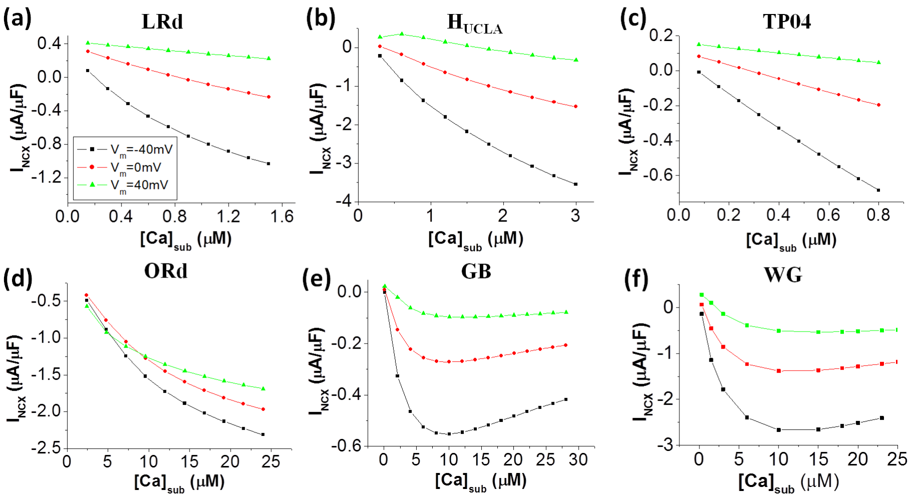

Fig D: Ca regulation on I_{NCX}. increasing $[Ca]_{sub}$ augments I_{NCX} thereby depolarizes V_m.

As for I_{Ks} which is also associated with $[Ca]_{sub}$, we find that in all the models $[Ca]_{sub}$'s regulation on it is small, as shown in Fig E. Therefore, we assume that I_{Ks} plays a small role in V_m-Ca coupling.

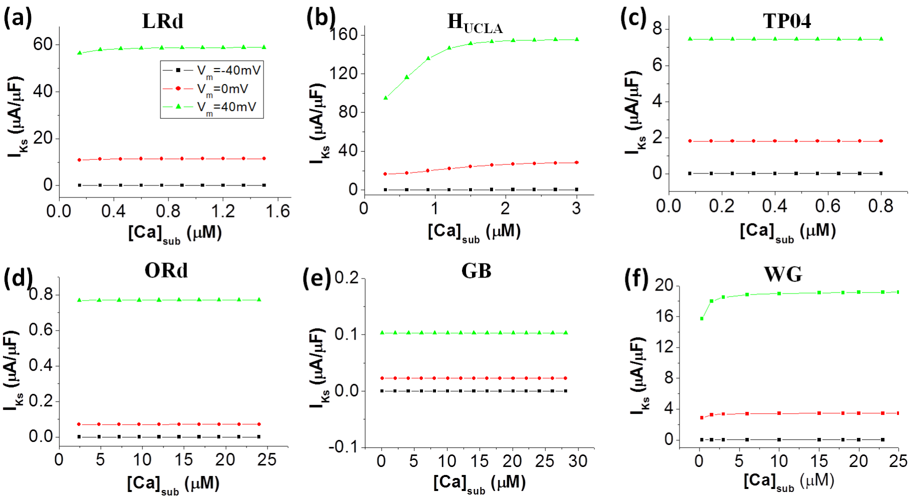

Fig E: Effects of Ca on the slow component of the delayed inward rectified potassium current (I_{Ks}). In all models, I_{Ks} exhibts a weak dependence on $[Ca]_{sub}$.

Types of EADs in different models

We investigate EAD behaviors in 5 other AP models besides the WG model. A Monte Carlo approach of randomly assigning parameters between 0.1-10 times of the control values is used to assess EADs. The selected random parameters and their control values for each model are stated in the parameter section below. For each model, a total of 10000 parameter sets is simulated, and the statistics are given in Table 1 in the main text. Typical EADs of different models are shown in Figs F-M. In the figures, unless specified otherwise, the parameters are set by the control ones as listed in the parameter section below. The parameter changes are given as times of the control ones (presented as α). Fig F is for the LRd model, Figs G and H are for the H_{UCLA} model, Fig I is for the TP04 model, Fig J is for the ORd model, and Figs K-M are for the GB model.

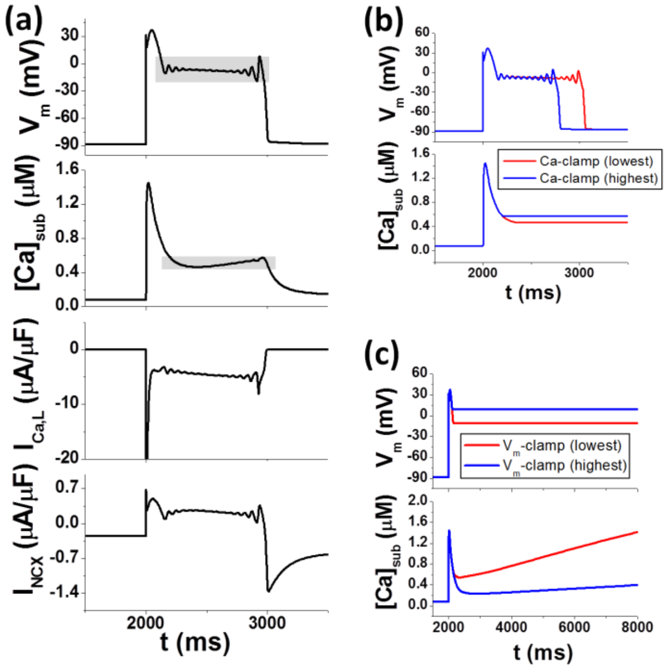

Fig F: V_m-driven EADs in the LRd model. (a) The original traces. The grey patches indicate the ranges for V_m and $[Ca]_{sub}$ clamping experiments. (b) Ca-clamp experiment. EADs occur when Ca is clamped. (c) V_m-clamp experiment. No $[Ca]_{sub}$ oscillation occurs.

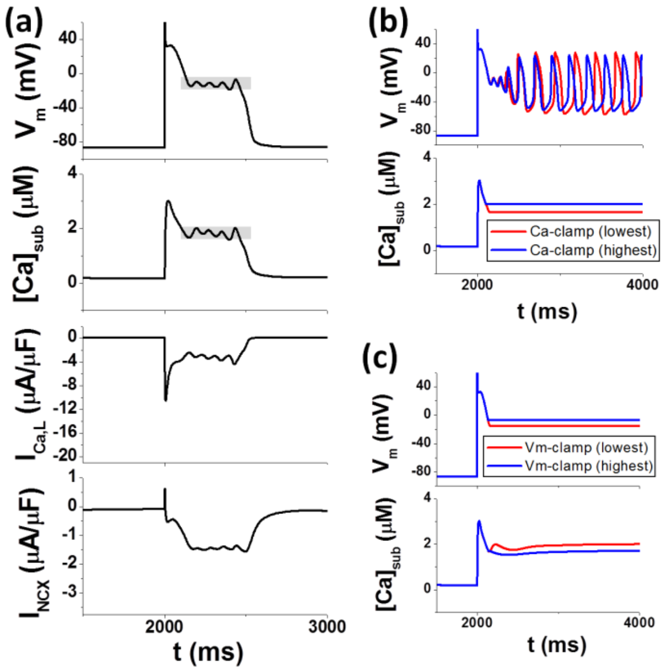

Fig G: V_m-driven EADs in the H_{UCLA} model. (a) The original traces. (b) The Ca-clamp experiment. (c) The V_m-clamp experiment.

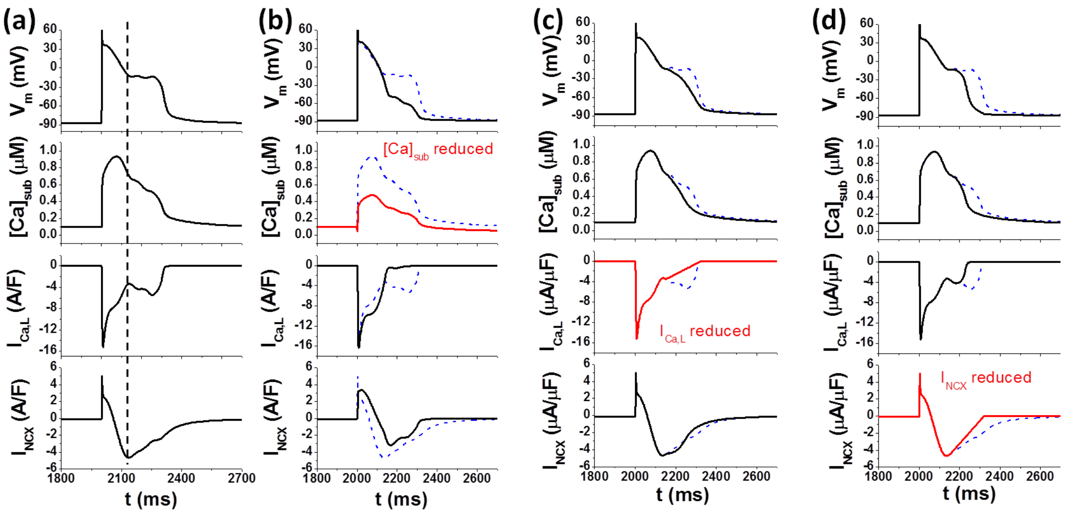

Fig H: Ca transient-driven EADs in the H_{UCLA} model. α(G_{CaL})=0.684, α(G_{Ks})=2.84, α(G_{ncx})=9.63, α(g_{RyR})=2.42, α(τ_s)=9.42, and α(τ_r)=1.0. (a) The original traces. (b) The scenario of $[Ca]_{sub}$ reduction ($[Ca]_{sub}$'s wave form is kept but the value is overall reduced by 0.5 times). The blue dashed traces are the original ones for comparison. (c) The scenario of $I_{Ca,L}$ reduction. Starting from the EAD takeoff moment, $I_{Ca,L}$ is forced to linearly decrease with time. (d) The scenario of I_{NCX} reduction (also linearly reduced from the EAD takeoff moment). Scenarios of (c) and (d) illustrate that this kind of EAD is pulled up by the joint work of $I_{Ca,L}$ and I_{NCX} under sufficient intracellular Ca. The intensity of I_{NCX} depends on the level of Ca transient so that reduced Ca transient attenuates EADs via reduction of I_{NCX}.

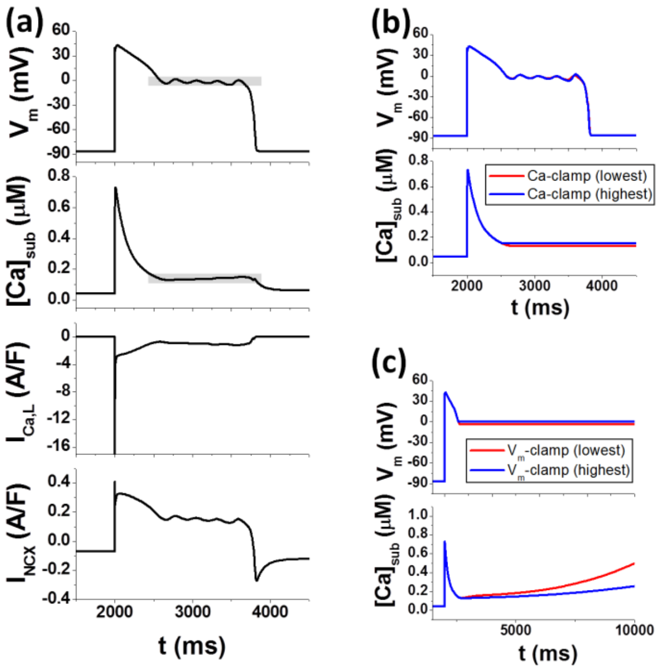

Fig I: V_m-driven EADs in the TP04 model. (a) The original traces. (b) The Ca-clamp experiment. (c) The V_m-clamp experiment. The properties are similar to the LRd model.

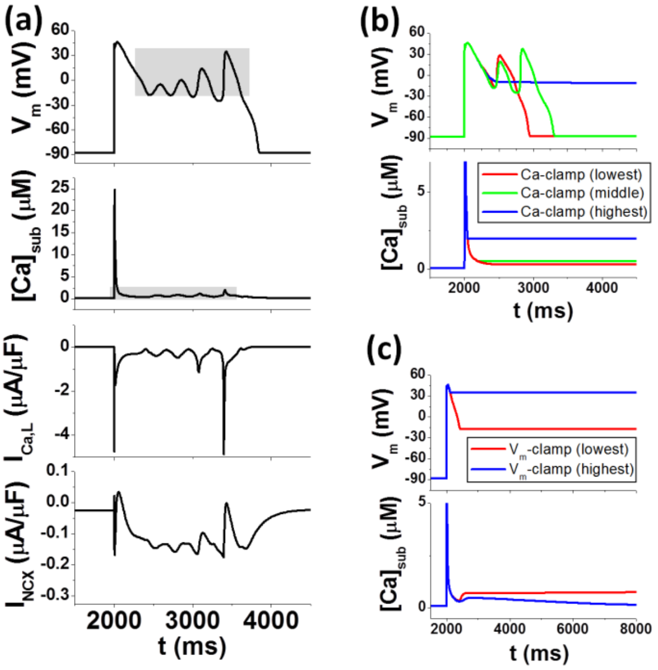

Fig J: V_m-driven EADs in the ORd model. (a) The original traces. (b) The Ca-clamp experiment. EAD dynamics in this model is very sensitive to $[Ca]_{sub}$, but EADs are generated inherently by the V_m system. (c) The V_m-clamp experiment.

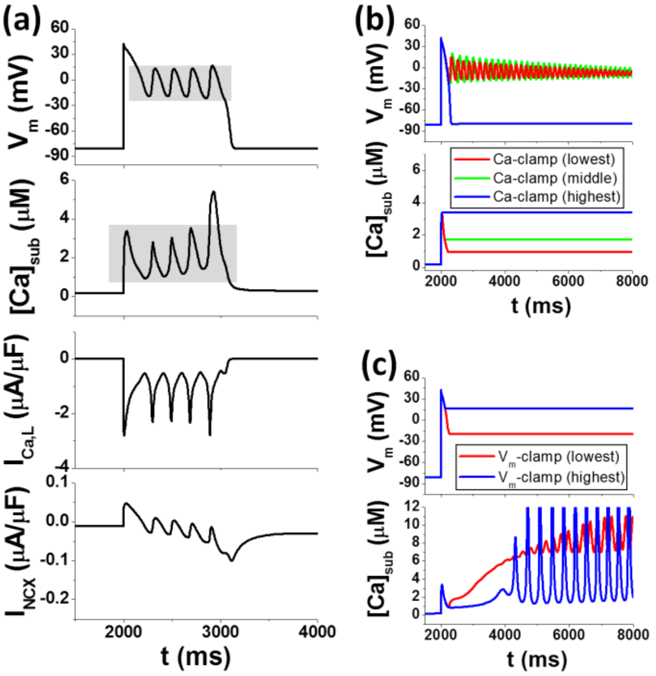

Fig K: V_m-driven EADs in the GB model. (a) The original traces. (b) The Ca-clamp experiment. V_m system is oscillatory in Ca clamp. (c) The V_m-clamp experiment. Different to the other models, $[Ca]_{sub}$ may oscillate undre V_m clamp.

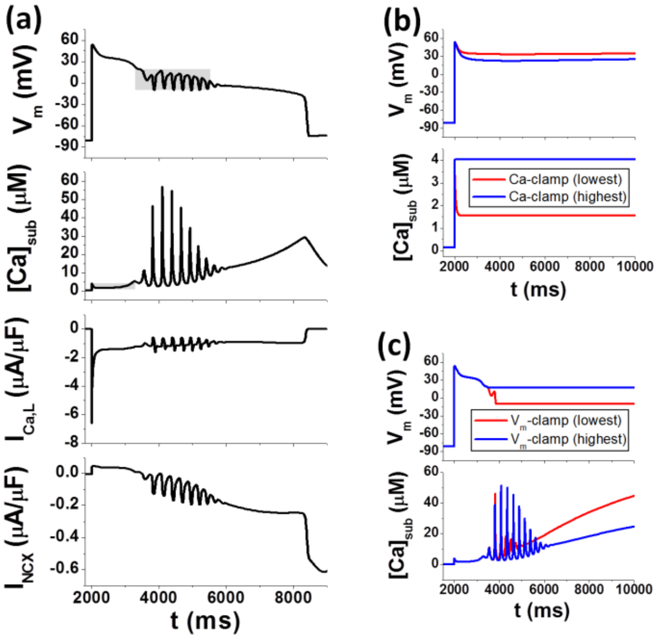

Fig L: Ca oscillation-driven EADs in the GB model. α(P_{Ca})=3.58, α(G_{Ks})=1.28, α(G_{ncx})=0.75, α(k_s)=0.34, and α($J_{Caslmyo}$)=5.23. (a) The original traces. (b) The Ca-clamp experiment. No V_m oscillation exhibits. (c) The V_m-clamp experiment. Ca oscillations exhibit. Therefore, this type of EADs is driven by Ca oscillations.

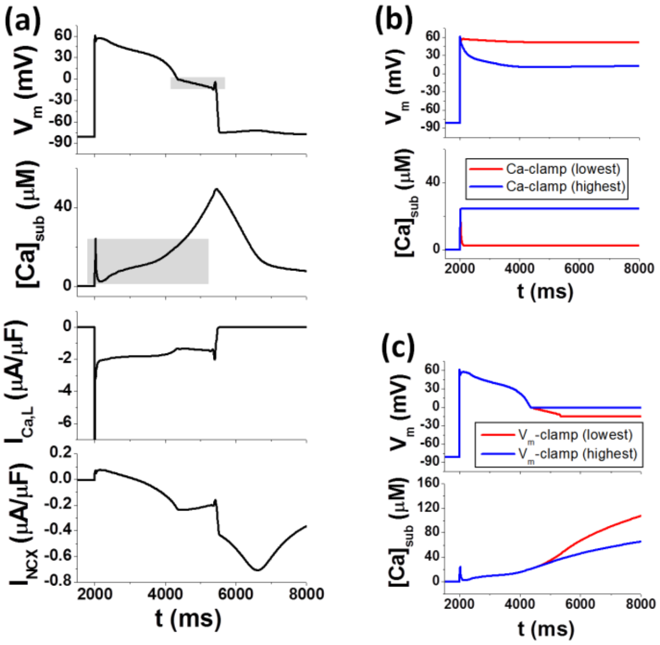

Fig M: EADs driven by the Ca-$I_{Ca,L}$-V_m-Ca feedback loop in the GB model. α(P_{Ca})=9.55, α(G_{Ks})=5.39, α(G_{ncx})=0.88, α(k_s)=1.63, and α($J_{Caslmyo}$)=3.95. (a) The original traces. (b) The Ca-clamp experiment. No V_m oscillation exhibits. (c) The V_m-clamp experiment. No Ca oscillation exhibits. This type of EAD is generated by the interplay between Ca and V_m.

Control parameter sets and initial conditions of the models

The control values of the parameters and initial conditions of the models are listed below.

1. The LRd model

Parameters: P_{Ca}=0.002 cm/ms, G_{Ks}=0.4 mS/μF, G_{ncx}=0.00025 μA/μF, g_{maxrel}=150 ms^{-1}, τ_{on}=0.5 ms, and τ_{off}=0.5 ms.

Stimulus: I_{sti}=80 μA/μF and duration=0.5 ms.

Initial conditions:

i) Membrane voltage and gating variables: V_m=-88.654973 mV, m=0.0008, h=0.9933, j=0.9955, d=0, f=0.9997, X_{s1}=0.0045, X_{s2}=0.0045, and X_r=0.0001;

ii) Intracellular concentrations and buffering (mM): $[Na^+]_i$=12.2364, $[Na^+]_o$=140, $[K^+]_i$=136.8915, $[K^+]_o$=4.5, $[Ca^{2+}]_i$=0.00002, $[Ca^{2+}]_o$=1.8, $[Ca^{2+}]_{jsr}$=1.17999, $[Ca^{2+}]_{nsr}$=1.17999, troponin=0.0144, and calsequestrin=6.9798.

2. The H_{UCLA} model

Parameters: G_{CaL}=300 mM/(cm, C), G_{Ks}=0.9 mS/μF, G_{ncx}=1.0 μM/s, g_{RyR}=2.58 cm^2/mA, τ_s=0.5 ms, and τ_r=30 ms.

Stimulus: I_{sti}=40 μA/μF and duration=2 ms.

Initial conditions:

i) Membrane voltage and gating variables: V_m=-86.7955 mV, m=0.0011, h=0.9898, j=0.9931, d=0, f=1, f_{Ca}=1, X_{s1}=0.0843, X_{s2}=0.1413, X_r=0.0087, $X_{to,f}$ =0.0037, $Y_{to,f}$=0.9824, $X_{to,s}$=0.0038, and $Y_{to,s}$=0.1553;

ii) Intracellular concentrations and buffering (μM): C_p=1.6826, C_s=2.0163, C_i=0.3864, C_j=107.0389, C_j'=95.7626, $[Na^+]_i$=8.0, J_{rel}=0.0065 μM/ms, $J^i_{troponin}$=29.6481 μM/ms, and $J^s_{troponin}$=26.3773 μM/ms.

3. The TP04 model

Parameters: G_{CaL}=0.0008 cm^3/(μF s), G_{Ks}=0.072 nS/pF, G_{ncx}=1000 pA/pF, and g_{rel}=0.016464 mM/ms.

Stimulus: I_{sti}=52 pA/pF and duration=1 ms.

Initial conditions:

i) Membrane voltage and gating variables: V_m =-86.2 mV, m=0, h=0.75, j=0.75 d=0, f=1, f_{Ca}=1, X_s=0, X_{r1}=0, X_{r2}=1, r=0, s=1, and g=1;

ii) Intracellular concentrations and buffering (mM): $[Ca^{2+}]_i$=0.00008, $[Ca^{2+}]_{SR}$=0.2, $[Na^+]_i$=11.6, and $[K^+]_i$=138.3.

4. The ORd model

Parameters: P_{Ca}=0.0003 cm/s, G_{Ks}=0.01 mS/μF, G_{ncx}=0.0008 μA/μF, α_{rel}=$\alpha_{rel,CaMK}$=2.375 ms, and β_τ=4.75 ms.

Stimulus: I_{sti}=80 μA/μF, duration=0.5 ms.

Initial conditions:

i) Membrane voltage and gating variables: V_m = -88.3022 mV, m=0.0071, h_{fast}=0.7084, h_{slow}=0.7084, j=0.7083, $h_{CaMK,slow}$=0.4672, j_{CaMK}=0.7082, m_L=1.78e-4, h_L=0.5121, $h_{L,CaMK}$=0.2801, a=9.81e-4, i_{fast}=0.9996, i_{slow}=0.5995, a_{CaMK}=5e-4, $i_{CaMK,fast}$=0.9996,

$i_{CaMK,slow}$=0.6549, d=0, f_{fast}=1, f_{slow}=0.9183, $f_{Ca,fast}$=1, $f_{Ca,slow}$=0.9999, j_{Ca}=1, n=0.0029, $f_{CaMK,fast}$=1, $f_{Ca,CaMK,fast}$=1, X_{s1}=0.2551, X_{s2}=1.86e-4, $X_{r,fast}$ =8e-6, $X_{r,slow}$=0.4391, and X_{K1}=0.9967;

ii) Intracellular concentrations and fluxes (mM): $[Na^+]_i$=7.0, $[Na^+]_{ss}$=7.0, $[K^+]_i$=145.4175, $[K^+]_{ss}$=145.41751, $[Ca^{2+}]_i$=8.7e-5, $[Ca^{2+}]_{ss}$=8.7e-5, $[Ca^{2+}]_{nsr}$=1.6553, $[Ca^{2+}]_{jsr}$=1.6083, $J_{rel,NP}$=0 mM/ms, $J_{rel,CaMK}$=0 mM/ms, and $CaMK_{trap}$=0.01311.

5. The GB model

Parameters: P_{Ca}=0.0001725 cm/s, G_{Ks}=0.001 mS/μF, G_{ncx}=0.9225 μA/μF, ks=0.2 ms^{-1}, and $J_{Caslmyo}$=3.7243×10^{-12} l/ms.

Stimulus: I_{sti}=50 μA/μF, duration=1 ms.

Initial conditions:

i) Membrane voltage and gating variables: V_m=-80.9763 mV, m=1.4e-3, h=0.9867, j=0.9916, d=7.2e-6, f=1, f_{CaBj}=0.0242, f_{CaBsl}=0.0145, X_{Ks}=0.0054, X_{Kr}=0.0086, $X_{to,s}$ = 0.0041, $Y_{to,s}$=0.9946, $X_{to,f}$=0.0041, and $Y_{to,f}$=0.9946;

ii) RyR variables (the state of RyR): R=0.8884, O=8.2e-7, and I=1e-7;

iii) Buffering variables (mM): Na_{Bj}=3.5399, Na_{Bsl}=0.7721, TnC_l=0.0088, TnC_{hc}=0.1078, TnC_{hm}=0.0152, CaM=2.9e-4, Myosin (Ca)=1.3e-3, Myosin (Mg)=0.1382, SRB=2.1e-3, SLL_j=9.6e-3, SLL_{sl}=0.111, SLH_j=7.3e-3, SLH_{sl}=0.073, and $Csqn_b$=1.243;

iv) Intracellular concentrations (mM): $[Ca^{2+}]_{sr}$=0.01, $[Ca^{2+}]_j$=1.7e-4, $[Ca^{2+}]_{sl}$=1e-4, $[Ca^{2+}]_i$=8.6e-5, $[Na^+]_j$=9.06, $[Na^+]_{sl}$=9.06, $[Na^+]_i$= 9.06, and $[K^+]_i$=120.0.

6. The WG model

Parameters: P_{Ca}=0.001188 cm/s, G_{Ks}=0.41 mS/μF, $\bar{I}_{NCX}$=5 μA/μF, k_{max}=0.2 ms^{-1}, and $J_{Caslmyo}$=7.4485×10^{-13} l/ms.

Stimulus: I_{sti}=40 pA/pF and duration=1 ms.

Initial conditions:

i) Membrane voltage and gating variables: V_m=-85.848 mV, m=0.0013, h=0.9875, j=0.992, d=0.0, f=1.0, f_{CaBj}=0.0367, f_{CaBsl}=0.0312, X_{Kr}=0.0083, X_{Ks}=0.0053, $X_{to,s}$=0.004, $Y_{to,s}$=0.4888, $R_{to,s}$=0.5259, $X_{to,f}$=0.004, and $Y_{to,f}$=0.9947;

ii) RyR variables (the state of RyR): O_1=0.0001, O_2=0.7131, and O_3=0.9935;

iii) Buffering variables (mM): Na_{Bj}=3.7413, Na_{Bsl}=0.8164, TnC_l=0.0082, TnC_{hc}=0.1217, TnC_{hm}=0.0085, CaM=0.0003, Myosin (Ca)=0.0022, Myosin (Mg)=0.1373, SRB=0.002, SLL_j=0.0112, SLL_{sl}=0.0209, SLH_j=0.0939, SLH_{sl}=0.07, $Csqn_b$=2.1384, and $Csqn_{nsr}$= $Csqn_{jsr}$ =2.1929;

iv) Intracellular concentrations (mM): $[Ca^{2+}]_{jsr}$=0.454, $[Ca^{2+}]_{jxn}$=0.0003, $[Ca^{2+}]_{sl}$=0.0002, $[Ca^{2+}]_i$=0.0001, $[Ca^{2+}]_{nsr}$=0.474, $[Na^+]_j$=9.7727, $[Na^+]_{sl}$=9.773, $[Na^+]_i$=9.7737, and $[K^+]_i$=135.0.
