# Supplementary material for: Dissecting the roles of calcium cycling and its coupling with voltage in the genesis of early afterdepolarizations in cardiac myocyte models
Source: PLoS Comput Biol. 2024 Feb 28;20(2):e1011930. doi: 10.1371/journal.pcbi.1011930 (PMC10927084; doi:10.1371/journal.pcbi.1011930)
Supplement: S1 Code — (DOCX) [file pcbi.1011930.s002.docx]

!--------------------------------------------------------------------------------------------------------------------------------

!This code is for the simulations of the WG model, written in fortran by Rui Wang.

!--------------------------------------------------------------------------------------------------------------------------------

module para

real*8::taug3,R,Frdy,Temp,FoRT,Cmem,Qpow

common taug3,R,Frdy,Temp,FoRT,Cmem,Qpow

real*8::cellLength,cellRadius,Vcell,Vmyo,Vsr,Vsl,Vjunc

common cellLength,cellRadius,Vcell,Vmyo,Vsr,Vsl,Vjunc

real*8::J_ca_juncsl,J_ca_slmyo,J_na_juncsl,J_na_slmyo,Fjunc,Fsl,Fjunc_CaL,Fsl_CaL,Cli,Clo,Ko,Nao,Cao,Mgi,ena_junc,ena_sl,ek,eca_junc,eca_sl,ecl

common J_ca_juncsl,J_ca_slmyo,J_na_juncsl,J_na_slmyo,Fjunc,Fsl,Fjunc_CaL,Fsl_CaL,Cli,Clo,Ko,Nao,Cao,Mgi,ena_junc,ena_sl,ek,eca_junc,eca_sl,ecl

real*8::GNa,GNaB,IbarNaK,KmNaip,KmKo,Q10NaK,Q10KmNai,pNaK,gkp,GtoSlow,GtoFast,GClCa,GClB,KdClCa,pNa,pCa,pK,Q10CaL,IbarNCX

common GNa,GNaB,IbarNaK,KmNaip,KmKo,Q10NaK,Q10KmNai,pNaK,gkp,GtoSlow,GtoFast,GClCa,GClB,KdClCa,pNa,pCa,pK,Q10CaL,IbarNCX

real*8::KmCai,KmCao,KmNai,KmNao,ksat,nu,Kdact,Q10NCX,IbarSLCaP,KmPCa,GCaB,Q10SLCaP,Q10SRCaP,Vmax_SRCaP

common KmCai,KmCao,KmNai,KmNao,ksat,nu,Kdact,Q10NCX,IbarSLCaP,KmPCa,GCaB,Q10SLCaP,Q10SRCaP,Vmax_SRCaP

real*8::Kmf,Kmr,hillSRCaP,ks,K1,K2,H1,H2,H3,B,A

common Kmf,Kmr,hillSRCaP,ks,K1,K2,H1,H2,H3,B,A

real*8::dif,nsrv,taug1,taug2,Bmax_Naj,Bmax_Nasl,koff_na,kon_na,Bmax_TnClow,koff_tncl,kon_tncl,Bmax_TnChigh,koff_tnchca,kon_tnchca,koff_tnchmg,kon_tnchmg,Bmax_CaM,koff_cam,kon_cam,Bmax_myosin,koff_myoca,kon_myoca,koff_myomg,kon_myomg,Bmax_SR,koff_sr,kon_sr,Bmax_SLlowsl,Bmax_SLlowj ,koff_sll,kon_sll,Bmax_SLhighsl,Bmax_SLhighj,koff_slh,kon_slh,Bmax_Csqn,koff_csqn,kon_csqn

common dif,nsrv,taug1,taug2,Bmax_Naj,Bmax_Nasl,koff_na,kon_na,Bmax_TnClow,koff_tncl,kon_tncl,Bmax_TnChigh,koff_tnchca,kon_tnchca,koff_tnchmg,kon_tnchmg,Bmax_CaM,koff_cam,kon_cam,Bmax_myosin,koff_myoca,kon_myoca,koff_myomg,kon_myomg,Bmax_SR,koff_sr,kon_sr,Bmax_SLlowsl,Bmax_SLlowj ,koff_sll,kon_sll,Bmax_SLhighsl,Bmax_SLhighj,koff_slh,kon_slh,Bmax_Csqn,koff_csqn,kon_csqn

real*8::dtt,T

real*8::ydot(45)

real*8::ICaL,INCX,ICajunc,ICasl,Incxjunc,Incxsl,T1,T2

common ICaL,INCX,ICajunc,ICasl,Incxjunc,Incxsl,T1,T2

real*8::numslmyo,numkmax,numpCa,numks,numserca,numncx ! Random parameter multiples

real*8::alpha_tauf,alpha_pCa,alpha_ncx,alpha_nak,alpha_gtos,alpha_gtof,alpha_gks,alpha_gkr,alpha_gki,alpha_nal,alpha_slmyo,alpha_sr

integer::LCC_manipulate,NCX_manipulate,Ca_manipuate,V_manipulate

common LCC_manipulate,NCX_manipulate,Ca_manipulate,V_manipulate

end module

!-----------------------------------------------------------------------------------------

! Gating variables corresponding to y(1:45)

! 1 2 3 4 5 6 7 8 9 10 11 12 13

!! m h j d f fcaBj fcaBsl xtos ytos xtof ytof xkr xks

!-----------------------------------------------------------------------------------------

! RyR and Buffering variables

! 14 15 16 17 18 19 20 21 22 23 24

!! RyRr RyRo RyRi NaBj NaBsl TnCL TnCHc TnCHm CaM Myoc Myom

!-----------------------------------------------------------------------------------------

! More buffering variables

! 25 26 27 28 29 30

!! SRB SLLj SLLsl SLHj SLHsl Csqnb

!----------------------------------------------------------------------------------------

! Intracellular concentrations/ Membrane voltage

! 31 32 33 34 35 36 37 38 39 40

! ! Ca_sr Naj Nasl Nai Ki Caj Casl Cai Vm rtos

!----------------------------------------------------------------------------------------

! NSR variables

! 41 42 43 44 45

! ! Ca_nsr Csqnb_NSR ryrg1 ryrg2 ryrg3

!----------------------------------------------------------------------------------------

program main

use para

implicit none

integer,parameter::step=3000000 ! step number

real*8::y(45)

integer::nn,tt,nnn,ii,jj,xn,xi,yi,zi

real*8::V_Clamp,Ca_junc_Clamp,Ca_sl_Clamp

real*8::LCC_junc_Clamp,LCC_sl_Clamp,NCX_junc_Clamp,NCX_sl_Clamp ! Record of corresponding values during clamping

dtt=0.002 ! Set step size during calculation

T=0.0

V_manipulate=0; Ca_manipulate=0; LCC_manipulate=0; NCX_manipulate=0; ! Clamp options (1=open 0=close)

open(12,file="action potential.dat",status="unknown")

! Random parameter multiples

numpCa=5.4;numks=1.16;numslmyo=2.64;numserca=1;numkmax=2;numncx=1; ! FIG2 Vm-oscillation

!numpCa=2.8;numks=0.56666;numslmyo=3.6;numserca=1;numkmax=7;numncx=2.2; ! FIG3 Ca-oscillation-NCX

!numpCa=8.2;numks=1.6;numslmyo=2.64;numserca=1;numkmax=2;numncx=1; ! FIG4 Ca-load-LCC

!numpCa=1;numks=1;numslmyo=1.2;numserca=1;numkmax=7;numncx=1; ! FIG5 Ca-transient-Vm

T1=415; ! Clamp time set up

T2=T1+0.002

! Variable initial value

y(1:45)=(/0.0013,0.9875,0.9920,0.0000,1.0014,0.0367,0.0312,0.0040,0.4888,0.0040,0.9947,&

0.0083,0.0053,0.8884,0.0,0.0,3.7413,0.8164,0.0082,0.1217,0.0085,0.0003,0.0022,0.1373,&

0.0020,0.0112,0.0209,0.0939,0.0700,2.1384,0.4540,9.7727,9.7730,9.7737,135.0000,0.0003,&

0.0002,0.0001,-85.8480,0.5259,0.4740,2.1929,0.0001,0.7131,0.9935/)

alpha_pCa=1;

alpha_gks=1;

alpha_gkr=1;

alpha_gtos=1;

alpha_gtof=1;

alpha_gki=1;

alpha_ncx=1;

alpha_nak=1;

alpha_tauf=1;

alpha_nal=0;

alpha_slmyo=1

alpha_sr=1;

call set_para()

do tt=1,step

! Clamp V

if(V_manipulate==1)then

if(T>=T1.and.T<=T2)then ! Record of corresponding values during clamping

V_Clamp=y(39);

else if(T>T2)then

y(39)=V_Clamp; ! clamping

end if

end if

! Clamp Ca

if(Ca_manipulate==1)then

if(T>=T1.and.T<=T2)then

Ca_junc_Clamp=y(36);

Ca_sl_Clamp=y(37);

else if(T>T2)then

y(36)=Ca_junc_Clamp;y(37)=Ca_sl_Clamp

end if

end if

! Clamp LCC

if(LCC_manipulate==1)then

if(T>=T1.and.T<=T2)then

LCC_junc_Clamp=ICajunc;

LCC_sl_Clamp=ICasl;

else if(T>T2)then

icajunc= LCC_junc_Clamp;icasl=LCC_sl_Clamp

end if

end if

! Clamp NCX

if(NCX_manipulate==1)then

if(T>=T1.and.T<=T2)then

NCX_junc_Clamp=Incxjunc;

NCX_sl_Clamp=Incxsl;

else if(T>T2)then

Incxjunc=NCX_junc_Clamp;Incxsl=NCX_sl_Clamp

end if

end if

!write(12,'(f12.6,f12.6)')0.6*y(36),0.6*y(37) ！ FIG5 Record the decreased CA

!read(48,'(f12.6,f12.6)')ca_jxn,ca_sl ！FIG5 Read the decreased CA

!y(36)=ca_jxn;y(37)=ca_sl;

call update(nnn,y(1:45))

y=y+ydot*dtt

if(mod(tt,25)==0) write(12,'(f12.6,f12.6,f12.6,f12.6,f12.6)')T,y(39),1000*y(36),ICajunc+ICasl,Incxjunc+Incxsl ! action potential output

T=T+dtt

end do

end program

! RabbitSR Model

subroutine set_para()

use para

implicit none

real*8,parameter::pi=3.1415926

integer::n

! Model Parameters

taug3=300;

R = 8314; ! [J/kmol*K]

Frdy = 96485; ! [C/mol]

Temp = 310; ! [K]

FoRT = Frdy/R/Temp;

Cmem = 1.3810e-10; ! [F] membrane capacitance

Qpow = (Temp-310)/10;

! Cell geometry

cellLength = 100; ! cell length [um]

cellRadius = 10.25; ! cell radius [um]

Vcell = pi*cellRadius**2*cellLength*1e-15; ! [L]

Vmyo = 0.65*Vcell; Vsr = 0.035*Vcell; Vsl = 0.02*Vcell; Vjunc = 0.0539*.01*Vcell;

J_ca_juncsl = 1/1.2134e12; ! [L/msec] = 8.2413e-13

J_ca_juncsl = 8*J_ca_juncsl;

J_ca_slmyo = 1/2.68510e11*0.2; ! [L/msec] = 3.2743e-12

J_ca_slmyo = numslmyo*J_ca_slmyo;

J_na_juncsl = 1/(1.6382e12/3*100); ! [L/msec] = 6.1043e-13

J_na_slmyo = 1/(1.8308e10/3*100); ! [L/msec] = 5.4621e-11

! Fractional currents in compartments

Fjunc = 0.11; Fsl = 1-Fjunc;

Fjunc_CaL = 0.5; Fsl_CaL = 1-Fjunc_CaL;

! Fixed ion concentrations

Cli = 15; ! Intracellular Cl [mM]

Clo = 150; ! Extracellular Cl [mM]

Ko = 5.4; ! Extracellular K [mM]

Nao = 140; ! Extracellular Na [mM]

Cao = 1.8; ! Extracellular Ca [mM]

Mgi = 1; ! Intracellular Mg [mM]

! Na transport parameters

GNa=16;

GNaB = 0.297e-3; ! [mS/uF]

IbarNaK = 1.90719; ! [uA/uF]

KmNaip = 11; ! [mM]

KmKo = 1.5; ! [mM]

Q10NaK = 1.63;

Q10KmNai = 1.39;

IbarNaK=IbarNaK*alpha_nak;

!! K current parameters

pNaK = 0.01833;

GtoSlow = 0.06*1; ! [mS/uF] !0.09 CaMKII

GtoFast = 0.02*1; ! [mS/uF]

gkp = 0.001;

GtoSlow=GtoSlow*alpha_gtos;

GtoFast=GtoFast*alpha_gtof;

! Cl current parameters

GClCa = 0.109625; ! [mS/uF]

GClB = 9e-3; ! [mS/uF]

KdClCa = 100e-3; ! [mM]

! I_Ca parameters

pNa = 1.5e-8; ! [cm/sec]

pCa = 5.4e-4*0.5; ! [cm/sec]

pCa = numpCa*pCa;

pCa = pCa*alpha_pCa;

pK = 2.7e-7; ! [cm/sec]

Q10CaL = 1.8;

! Ca transport parameters

IbarNCX = 5;

KmCai = 3.59e-3; ! [mM]

KmCai = KmCai;

KmCao = 1.3; ! [mM]

KmNai = 12.29; ! [mM]

KmNao = 87.5; ! [mM]

ksat = 0.27; ! [none]

nu = 0.35; ! [none]

Kdact = 0.256e-3; ! [mM]

Q10NCX = 1.57; ! [none]

IbarSLCaP = 0.0673; ! [uA/uF](2.2 umol/L cytosol/sec)

KmPCa = 0.5e-3; ! [mM]

GCaB = 2.513e-4; ! [uA/uF]

Q10SLCaP = 2.35; ! [none]

! SR flux parameters

Q10SRCaP = 2.6; ! [none]

Vmax_SRCaP = 5.3114e-3; ! [mM/msec] (286 umol/L cytosol/sec)

Vmax_SRCaP = 1*Vmax_SRCaP;

Kmf = 0.246e-3; ! [mM] default

Kmf = Kmf;

Kmr = 1.7; ! [mM]L cytosol

hillSRCaP = 1.787; ! [mM]

hillSRCaP = hillSRCaP;

ks = 5;

K1 = 0.01; ! [mM]

K2=0.35; ! [mM]

H1 = 2.7;

H2 = 3.5;

H3 = 4;

B = 0.004;

A=0.04;

dif = 0.01; ! [1/ms] diffusion coef NSR-JSR

nsrv = 1; ! [none] NSR volume / JSR volume

taug1=20; ![ms]

! Buffering parameters

! Note: we are using [1/ms] and [1/mM/ms], which differs from that in the paper

! koff: [1/s] = 1e-3*[1/ms]; kon: [1/uM/s] = [1/mM/ms]

Bmax_Naj = 7.561; ! [mM] ! Na buffering

Bmax_Nasl = 1.65; ! [mM]

koff_na = 1e-3; ! [1/ms]

kon_na = 0.1e-3; ! [1/mM/ms]

Bmax_TnClow = 70e-3; ! [mM] ! TnC low affinity

koff_tncl = 19.6e-3; ! [1/ms]

kon_tncl = 32.7; ! [1/mM/ms]

Bmax_TnChigh = 140e-3; ! [mM] ! TnC high affinity

koff_tnchca = 0.032e-3; ! [1/ms]

kon_tnchca = 2.37; ! [1/mM/ms]

koff_tnchmg = 3.33e-3; ! [1/ms]

kon_tnchmg = 3e-3; ! [1/mM/ms]

Bmax_CaM = 24e-3; ! [mM] ! CaM buffering

koff_cam = 238e-3; ! [1/ms]

kon_cam = 34; ! [1/mM/ms]

Bmax_myosin = 140e-3; ! [mM] ! Myosin buffering

koff_myoca = 0.46e-3; ! [1/ms]

kon_myoca = 13.8; ! [1/mM/ms]

koff_myomg = 0.057e-3; ! [1/ms]

kon_myomg = 0.0157; ! [1/mM/ms]

Bmax_SR = 19*.9e-3; ! [mM] (Bers text says 47e-3) 19e-3

koff_sr = 60e-3; ! [1/ms]

kon_sr = 100; ! [1/mM/ms]

Bmax_SLlowsl = 37.4e-3*Vmyo/Vsl; ! [mM] ! SL buffering

Bmax_SLlowj = 4.6e-3*Vmyo/Vjunc*0.1; ! [mM]

koff_sll = 1300e-3; ! [1/ms]

kon_sll = 100; ! [1/mM/ms]

Bmax_SLhighsl = 5e-3*Vmyo/Vsl; ! [mM]

Bmax_SLhighj = 1.65e-3*Vmyo/Vjunc*0.1; ! [mM]

koff_slh = 30e-3; ! [1/ms]

kon_slh = 100; ! [1/mM/ms]

Bmax_Csqn = 140e-3*Vmyo/Vsr; ! [mM] ! Bmax_Csqn = 2.6; ! Csqn buffering

Bmax_Csqn = 2*Bmax_Csqn;

koff_csqn = 65; ! [1/ms]

kon_csqn = 100; ! [1/mM/ms]

end subroutine

subroutine update(n,ypre)

use para

implicit none

real*8::ypre(45)

integer::n

real*8::am,bm,ah,bh,aj,bj,hinf,jinf

real*8::block1,eks,fnak,gkr,gks_junc,gks_sl

real*8::I_kp_junc,I_kr,I_ks,I_ks_junc,I_ks_sl

real*8::I_na,I_na_junc,I_na_sl,I_nabk,I_nabk_junc,I_nabk_sl,I_nak,I_nak_junc,I_nak_sl

real*8::kp_kp,pcaks_junc,pcaks_sl,rkr,sigma,tauxr,tauxs,xrss,xsss

real*8::aki,bki,dss,fcacaj,fcacamsl,fss

real*8::I_clbk,I_clca,I_clca_junc,I_clca_sl,I_ki,I_kp,I_kp_sl,I_to,I_tof,I_tos

real*8::kiss,rtoss,taud,tauf,taurtos,tauxtof,tauxtos,tauytof,tauytos,xtoss,ytoss

real*8::I_ca,I_ca_junc,I_ca_sl,I_cabk,I_cabk_junc,I_cabk_sl,I_cak,I_cana,I_cana_junc,I_cana_sl,I_catot,I_ncx,I_ncx_junc,I_ncx_sl

real*8::ibarca_j,ibarca_sl,ibark,ibark_j,ibark_sl,J_srcarel,ka_junc,ka_sl

real*8::s1_junc,s1_sl,s2_junc,s2_sl,s3_junc,s3_sl

real*8::H4,I_ca_tot,I_ca_tot_junc,I_ca_tot_sl,I_cl_tot,I_k_tot,I_na_tot,I_na_tot_junc,I_na_tot_sl,I_tot

real*8::J_cab_cytosol,J_cab_sl,J_cab_juncion,J_nsr,J_serca

real*8::K4,mi,ryrg1ss,ryrg2ss,z1

real*8::ibarna_j,ibarna_sl,J_cab_junction,z

real*8,external::I_app

! Nernst Potentials

ena_junc = (1/FoRT)*log(Nao/ypre(32)); ! [mV]

ena_sl = (1/FoRT)*log(Nao/ypre(33)); ! [mV]

ek = (1/FoRT)*log(Ko/ypre(35)); ! [mV]

eca_junc = (1/FoRT/2)*log(Cao/ypre(36)); ! [mV]

eca_sl = (1/FoRT/2)*log(Cao/ypre(37)); ! [mV]

ecl = (1/FoRT)*log(Cli/Clo); ! [mV]

taug2=0.02*(1+1/(ypre(31)**H3)); ![ms]

!! Membrane Currents

! I_Na: Fast Na Current

am = 0.32*(ypre(39)+47.13)/(1-exp(-0.1*(ypre(39)+47.13)));

bm = 0.08*exp(-ypre(39)/11);

if (ypre(39) >= -40) then

ah = 0; aj = 0;

bh = 1/(0.13*(1+exp(-(ypre(39)+10.66)/11.1)));

bj = 0.3*exp(-2.535e-7*ypre(39))/(1+exp(-0.1*(ypre(39)+32)));

else

ah = 0.135*exp((80+ypre(39))/-6.8);

bh = 3.56*exp(0.079*ypre(39))+3.1e5*exp(0.35*ypre(39));

aj = (-1.2714e5*exp(0.2444*ypre(39))-3.474e-5*exp(-0.04391*ypre(39)))*(ypre(39)+37.78)/(1+exp(0.311*(ypre(39)+79.23)));

bj = 0.1212*exp(-0.01052*ypre(39))/(1+exp(-0.1378*(ypre(39)+40.14)));

end if

hinf=ah/(ah+bh);

jinf=aj/(aj+bj);

ydot(1) = am*(1-ypre(1))-bm*ypre(1);

ydot(2) = (hinf-ypre(2))*(ah+bh);

ydot(3) = (jinf-ypre(3))*(aj+bj);

I_Na_junc = Fjunc*GNa*ypre(1)**3*ypre(2)*ypre(3)*(ypre(39)-ena_junc);

I_Na_sl = Fsl*GNa*ypre(1)**3*ypre(2)*ypre(3)*(ypre(39)-ena_sl);

I_Na = I_Na_junc+I_Na_sl;

! I_nabk: Na Background Current

I_nabk_junc = Fjunc*GNaB*(ypre(39)-ena_junc);

I_nabk_sl = Fsl*GNaB*(ypre(39)-ena_sl);

I_nabk = I_nabk_junc+I_nabk_sl;

! I_nak: Na/K Pump Current

sigma = (exp(Nao/67.3)-1)/7;

fnak = 1/(1+0.1245*exp(-0.1*ypre(39)*FoRT)+0.0365*sigma*exp(-ypre(39)*FoRT));

I_nak_junc = Fjunc*IbarNaK*fnak*Ko /(1+(KmNaip/ypre(32))**4) /(Ko+KmKo);

I_nak_sl = Fsl*IbarNaK*Q10NaK*fnak*Ko /(1+(KmNaip/ypre(33))**4) /(Ko+KmKo);

I_nak = I_nak_junc+I_nak_sl;

! I_kr: Rapidly Activating K Current

gkr = 0.03*sqrt(Ko/5.4);

gkr = 1.5*gkr;

gkr = gkr*alpha_gkr;

block1=0.2;

xrss = 1/(1+exp(-(ypre(39)+50)/7.5));

tauxr = 1/(1.38e-3*(ypre(39)+7)/(1-exp(-0.123*(ypre(39)+7)))+6.1e-4*(ypre(39)+10)/(exp(0.145*(ypre(39)+10))-1));

ydot(12) = (xrss-ypre(12))/tauxr;

rkr = 1/(1+exp((ypre(39)+33)/22.4));

I_kr = block1*gkr*ypre(12)*rkr*(ypre(39)-ek);

! I_ks: Slowly Activating K Current

pcaks_junc = -log10(ypre(36))+3.0;

pcaks_sl = -log10(ypre(37))+3.0;

! gks_junc = 0.07*(0.057 +0.19/(1+ exp((-7.2+pcaks_junc)/0.6)));

! gks_sl = 0.07*(0.057 +0.19/(1+ exp((-7.2+pcaks_sl)/0.6)));

gks_junc = 0.41*(0.057 +0.19/(1+ exp((-7.2+pcaks_junc)/0.6)));! New Value for ISOPROTERENOL

gks_sl = 0.41*(0.057 +0.19/(1+ exp((-7.2+pcaks_sl)/0.6))); ! New Value for ISOPROTERENOL

eks = (1/FoRT)*log((Ko+pNaK*Nao)/(ypre(35)+pNaK*ypre(34)));

xsss = 1/(1+exp(-(ypre(39)-1.5)/16.7));

tauxs = 1/(7.19e-5*(ypre(39)+30)/(1-exp(-0.148*(ypre(39)+30)))+1.31e-4*(ypre(39)+30)/(exp(0.0687*(ypre(39)+30))-1));

ydot(13) = (xsss-ypre(13))/tauxs;

I_ks_junc = Fjunc*gks_junc*ypre(13)**2*(ypre(39)-eks);

I_ks_sl = Fsl*gks_sl*ypre(13)**2*(ypre(39)-eks);

I_ks = I_ks_junc+I_ks_sl;

I_ks = numks*3*I_ks*alpha_gks;

!I_kp: Plateau K current

kp_kp = 1/(1+exp((7.488-ypre(39))/5.98));

I_kp_junc = Fjunc*gkp*kp_kp*(ypre(39)-ek);

I_kp_sl = Fsl*gkp*kp_kp*(ypre(39)-ek);

I_kp = I_kp_junc+I_kp_sl;

!! I_to: Transient Outward K Current (slow and fast components)

xtoss = 1/(1+exp(-(ypre(39)+3.0)/15));

ytoss = 1/(1+exp((ypre(39)+33.5)/10));

rtoss = 1/(1+exp((ypre(39)+33.5)/10));

tauxtos = 9/(1+exp((ypre(39)+3.0)/15))+0.5;

tauytos = 3e3/(1+exp((ypre(39)+60.0)/10))+30;

taurtos = 2.8e3/(1+exp((ypre(39)+60.0)/10))+220; ! time-dependent gating variable

ydot(8) = (xtoss-ypre(8))/tauxtos;

ydot(9) = (ytoss-ypre(9))/tauytos;

ydot(40)= (rtoss-ypre(40))/taurtos; !time-dependent gating variable

I_tos = GtoSlow*ypre(8)*(ypre(9)+0.5*ypre(40))*(ypre(39)-ek); ! [uA/uF]

tauxtof = 3.5*exp(-ypre(39)*ypre(39)/30/30)+1.5;

tauytof = 20.0/(1+exp((ypre(39)+33.5)/10))+20.0;

ydot(10) = (xtoss-ypre(10))/tauxtof;

ydot(11) = (ytoss-ypre(11))/tauytof;

I_tof = GtoFast*ypre(10)*ypre(11)*(ypre(39)-ek);

I_to = I_tos + I_tof;

! I_ki: Time-Independent K Current

aki = 1.02/(1+exp(0.2385*(ypre(39)-ek-59.215)));

bki =(0.49124*exp(0.08032*(ypre(39)+5.476-ek)) + exp(0.06175*(ypre(39)-ek-594.31))) /(1 + exp(-0.5143*(ypre(39)-ek+4.753)));

kiss = aki/(aki+bki);

I_ki = 0.9*sqrt(Ko/5.4)*kiss*(ypre(39)-ek);

I_ki = 0.9*sqrt(Ko/5.4)*kiss*(ypre(39)-ek)*alpha_gki;

! I_ClCa: Ca-activated Cl Current, I_Clbk: background Cl Current

I_ClCa_junc = Fjunc*GClCa/(1+KdClCa/ypre(36))*(ypre(39)-ecl);

I_ClCa_sl = Fsl*GClCa/(1+KdClCa/ypre(37))*(ypre(39)-ecl);

I_ClCa = I_ClCa_junc+I_ClCa_sl;

I_Clbk = GClB*(ypre(39)-ecl);

!! I_Ca: L-type Calcium Current

! v_py=0; !(ypre(39)+v_py)

! dss = 1/(1+exp(-((ypre(39)+v_py)+23.5)/6));

! taud = dss*(1-exp(-((ypre(39)+v_py)+23.5)/6))/(0.035*((ypre(39)+v_py)+23.5));

! fss = 1/(1+exp(((ypre(39)+v_py)+42)/3.6))+0*1/(1+exp((45-(ypre(39)+v_py))/20));! modified

! tauf = alpha_tauf*1/(0.0197*exp( -(0.0337*((ypre(39)+v_py)+14.5))**2 )+0.02);

dss = 1/(1+exp(-(ypre(39)-0+23.5)/6));

taud = dss*(1-exp(-(ypre(39)-0+23.5)/6))/(0.035*(ypre(39)-0+23.5));

fss = 1/(1+exp((ypre(39)-0+42)/3.6))+1/(1+exp((45-ypre(39)+0)/20));

! fss = 1/(1+exp((ypre(39)-0+42)/3.6));

tauf = 1/(0.0197*exp( -(0.0337*(ypre(39)-0+14.5))**2 )+0.02);

tauf=tauf+(49.96-tauf)/(49.96-25.19)*25.19*(alpha_tauf-1);

ydot(4) = (dss-ypre(4))/taud;

ydot(5) = (fss-ypre(5))/tauf;

ydot(6) = 1.7*ypre(36)*(1-ypre(6))-0.012*ypre(6); ! fCa_junc

ydot(7) = 1.7*ypre(37)*(1-ypre(7))-0.012*ypre(7); ! fCa_sl

!fcaCaMSL= 0.1/(1+(0.01/ypre(37)));

!fcaCaj= 0.1/(1+(0.01/ypre(36)));

fcaCaMSL=0;

fcaCaj= 0;

ibarca_j = pCa*4*(ypre(39)*Frdy*FoRT) * (0.341*ypre(36)*exp(2*ypre(39)*FoRT)-0.341*Cao) /(exp(2*ypre(39)*FoRT)-1);

ibarca_sl = pCa*4*(ypre(39)*Frdy*FoRT) * (0.341*ypre(37)*exp(2*ypre(39)*FoRT)-0.341*Cao) /(exp(2*ypre(39)*FoRT)-1);

ibark = pK*(ypre(39)*Frdy*FoRT)*(0.75*ypre(35)*exp(ypre(39)*FoRT)-0.75*Ko) /(exp(ypre(39)*FoRT)-1);

ibarna_j = pNa*(ypre(39)*Frdy*FoRT) *(0.75*ypre(32)*exp(ypre(39)*FoRT)-0.75*Nao) /(exp(ypre(39)*FoRT)-1);

ibarna_sl = pNa*(ypre(39)*Frdy*FoRT) *(0.75*ypre(33)*exp(ypre(39)*FoRT)-0.75*Nao) /(exp(ypre(39)*FoRT)-1);

I_Ca_junc = (Fjunc_CaL*ibarca_j*ypre(4)*ypre(5)*((1-ypre(6))+fcaCaj)*Q10CaL**Qpow)*0.45*1;

I_Ca_sl = (Fsl_CaL*ibarca_sl*ypre(4)*ypre(5)*((1-ypre(7))+fcaCaMSL)*Q10CaL**Qpow)*0.45*1;

I_Ca = I_Ca_junc+I_Ca_sl;

I_CaK = (ibark*ypre(4)*ypre(5)*(Fjunc_CaL*(fcaCaj+(1-ypre(6)))+Fsl_CaL*(fcaCaMSL+(1-ypre(7))))*Q10CaL**Qpow)*0.45*1;

I_CaNa_junc = (Fjunc_CaL*ibarna_j*ypre(4)*ypre(5)*((1-ypre(6))+fcaCaj)*Q10CaL**Qpow)*0.45*1;

I_CaNa_sl = (Fsl_CaL*ibarna_sl*ypre(4)*ypre(5)*((1-ypre(7))+fcaCaMSL)*Q10CaL**Qpow)*.45*1;

I_CaNa = I_CaNa_junc+I_CaNa_sl;

I_Catot = I_Ca+I_CaK+I_CaNa;

if(LCC_manipulate==1)then

if(T<T1)then

ICajunc=I_Ca_junc;ICasl=I_Ca_sl

else if(T>=T1)then

I_Ca_junc=ICajunc;I_Ca_sl=ICasl

end if

else

ICajunc=I_Ca_junc;ICasl=I_Ca_sl

end if

! I_ncx: Na/Ca Exchanger flux

IbarNCX=IbarNCX*alpha_ncx;

Ka_junc = 1/(1+(Kdact/ypre(36))**3);

Ka_sl = 1/(1+(Kdact/ypre(37))**3);

s1_junc = exp(nu*ypre(39)*FoRT)*ypre(32)**3*Cao;

s1_sl = exp(nu*ypre(39)*FoRT)*ypre(33)**3*Cao;

s2_junc = exp((nu-1)*ypre(39)*FoRT)*Nao**3*ypre(36);

s3_junc = KmCai*Nao**3*(1+(ypre(32)/KmNai)**3) + KmNao**3*ypre(36)*(1+ypre(36)/KmCai)+KmCao*ypre(32)**3+ypre(32)**3*Cao+Nao**3*ypre(36);

s2_sl = exp((nu-1)*ypre(39)*FoRT)*Nao**3*ypre(37);

s3_sl = KmCai*Nao**3*(1+(ypre(33)/KmNai)**3) + KmNao**3*ypre(37)*(1+ypre(37)/KmCai)+KmCao*ypre(33)**3+ypre(33)**3*Cao+Nao**3*ypre(37);

I_ncx_junc = Fjunc*IbarNCX*Q10NCX**Qpow*Ka_junc*(s1_junc-s2_junc)/s3_junc/(1+ksat*exp((nu-1)*ypre(39)*FoRT));

I_ncx_junc =I_ncx_junc *numncx

I_ncx_sl = Fsl*IbarNCX*Q10NCX**Qpow*Ka_sl*(s1_sl-s2_sl)/s3_sl/(1+ksat*exp((nu-1)*ypre(39)*FoRT));

I_ncx_sl=I_ncx_sl*numncx

I_ncx = I_ncx_junc+I_ncx_sl;

if(NCX_manipulate==1)then

if(T<T1)then

Incxjunc=I_ncx_junc;Incxsl=I_ncx_sl

else if(T>=T1)then

I_ncx_junc=Incxjunc;I_ncx_sl=Incxsl

end if

else

Incxjunc=I_ncx_junc;Incxsl=I_ncx_sl

end if

! I_cabk: Ca Background Current

I_cabk_junc = Fjunc*GCaB*(ypre(39)-eca_junc);

I_cabk_sl = Fsl*GCaB*(ypre(39)-eca_sl);

I_cabk = I_cabk_junc+I_cabk_sl;

!! SR fluxes: Calcium Release, SR Ca pump, SR Ca leak

ydot(14) = 0; ! R

ydot(15) = 0;! O

ydot(16) = 0; ! I

J_SRCarel = numkmax*ks*A*ypre(44)*ypre(45)*(B+ypre(43))*(ypre(31)-ypre(36)); ! [mM/ms]

!J_SRCarel = ks*A*(B+(ypre(36)/K1)**H1/(1+(ypre(36)/K1)**H1))*(ypre(31)-ypre(36));

J_serca = Q10SRCaP**Qpow*Vmax_SRCaP*((ypre(38)/Kmf)**hillSRCaP-(ypre(41)/Kmr)**hillSRCaP)&

/(1+(ypre(38)/Kmf)**hillSRCaP+(ypre(41)/Kmr)**hillSRCaP); ! [mM/ms], NSR volume

J_serca=numserca*J_serca

! J_SRleak = 5.348e-6*(ypre(31)-ypre(36)); ! [mM/ms]

J_NSR = nsrv * dif * (ypre(41)-ypre(31)); ! [mM/ms], from NSR to JSR is positive, JSR volume

!! Sodium and Calcium Buffering

ydot(17) = kon_na*ypre(32)*(Bmax_Naj-ypre(17))-koff_na*ypre(17); ! NaBj [mM/ms]

ydot(18) = kon_na*ypre(33)*(Bmax_Nasl-ypre(18))-koff_na*ypre(18); ! NaBsl [mM/ms]

! Cytosolic Ca Buffers

! ydot(19) = nc*(ydot(41)+ydot(42)+ydot(43));

ydot(19) = kon_tncl*ypre(38)*(Bmax_TnClow-ypre(19))-koff_tncl*ypre(19); ! TnCL [mM/ms]

ydot(20) = kon_tnchca*ypre(38)*(Bmax_TnChigh-ypre(20)-ypre(21))-koff_tnchca*ypre(20); ! TnCHc [mM/ms]

ydot(21) = kon_tnchmg*Mgi*(Bmax_TnChigh-ypre(20)-ypre(21))-koff_tnchmg*ypre(21); ! TnCHm [mM/ms]

ydot(22) = kon_cam*ypre(38)*(Bmax_CaM-ypre(22))-koff_cam*ypre(22); ! CaM [mM/ms]

ydot(23) = kon_myoca*ypre(38)*(Bmax_myosin-ypre(23)-ypre(24))-koff_myoca*ypre(23); ! Myosin_ca [mM/ms]

ydot(24) = kon_myomg*Mgi*(Bmax_myosin-ypre(23)-ypre(24))-koff_myomg*ypre(24); ! Myosin_mg [mM/ms]

ydot(25) = kon_sr*ypre(38)*(Bmax_SR-ypre(25))-koff_sr*ypre(25); ! SRB [mM/ms]

J_CaB_cytosol = sum(ydot(19:25));

! Junctional and SL Ca Buffers

ydot(26) = kon_sll*ypre(36)*(Bmax_SLlowj-ypre(26))-koff_sll*ypre(26); ! SLLj [mM/ms]

ydot(27) = kon_sll*ypre(37)*(Bmax_SLlowsl-ypre(27))-koff_sll*ypre(27); ! SLLsl [mM/ms]

ydot(28) = kon_slh*ypre(36)*(Bmax_SLhighj-ypre(28))-koff_slh*ypre(28); ! SLHj [mM/ms]

ydot(29) = kon_slh*ypre(37)*(Bmax_SLhighsl-ypre(29))-koff_slh*ypre(29); ! SLHsl [mM/ms]

J_CaB_junction = ydot(26)+ydot(28);

J_CaB_sl = ydot(27)+ydot(29);

!! Ion concentrations

! SR Ca Concentrations

ydot(30) = kon_csqn*ypre(31)*(Bmax_Csqn-ypre(30))-koff_csqn*ypre(30); ! Csqn [mM/ms]

ydot(31) = J_NSR-J_SRCarel-ydot(30); ! Ca_sr [mM/ms] !Ratio 3 leak current

ydot(42) = kon_csqn*ypre(41)*(Bmax_Csqn-ypre(42))-koff_csqn*ypre(42); ! Csqn conc in JSR and NSR assumed same

ydot(41) = J_serca-J_NSR-ydot(42);

ryrg1ss=(ypre(36)/K1)**H1/(1+(ypre(36)/K1)**H1);

ryrg2ss=(ypre(31)/K2)**H2/(1+(ypre(31)/K2)**H2);

ydot(43)=(ryrg1ss-ypre(43))/taug1;

ydot(44)=(ryrg2ss-ypre(44))/taug2;

!gate 3 - RyR inactivation d/t high junctional space Ca2+

K4 = 0.005;

H4 = 2;

mi = 1; !maximum block

z= (ypre(36)/K4)**H4/(1+(ypre(36)/K4)**H4);

z=1-mi*z;

ydot(45)= (z-ypre(45))/taug3;

! Sodium Concentrations

I_Na_tot_junc = I_Na_junc+I_nabk_junc+3*I_ncx_junc+3*I_nak_junc+I_CaNa_junc; ! [uA/uF]

I_Na_tot_sl = I_Na_sl+I_nabk_sl+3*I_ncx_sl+3*I_nak_sl+I_CaNa_sl; ! [uA/uF]

ydot(32) = -I_Na_tot_junc*Cmem/(Vjunc*Frdy)+J_na_juncsl/Vjunc*(ypre(33)-ypre(32))-ydot(17);

ydot(33) = -I_Na_tot_sl*Cmem/(Vsl*Frdy)+J_na_juncsl/Vsl*(ypre(32)-ypre(33))&

+J_na_slmyo/Vsl*(ypre(34)-ypre(33))-ydot(18);

ydot(34) = J_na_slmyo/Vmyo*(ypre(33)-ypre(34)); ! [mM/msec]

! Potassium Concentration

I_K_tot = I_to+I_kr+I_ks+I_ki-2*I_nak+I_CaK+I_kp; ! [uA/uF]

ydot(35) = 0; !-I_K_tot*Cmem/(Vmyo*Frdy); ! [mM/msec]

! Calcium Concentrations

I_Ca_tot_junc = I_Ca_junc+I_cabk_junc-2*I_ncx_junc; ! [uA/uF]

I_Ca_tot_sl = I_Ca_sl+I_cabk_sl-2*I_ncx_sl; ! [uA/uF]

ydot(36) = -I_Ca_tot_junc*Cmem/(Vjunc*2*Frdy)+J_ca_juncsl/Vjunc*(ypre(37)-ypre(36))&

-J_CaB_junction+(J_SRCarel)*Vsr/Vjunc; ! Ca_junc

ydot(37) = -I_Ca_tot_sl*Cmem/(Vsl*2*Frdy)+J_ca_juncsl/Vsl*(ypre(36)-ypre(37))&

+ J_ca_slmyo/Vsl*(ypre(38)-ypre(37))-J_CaB_sl; ! Ca_sl

if(Ca_manipulate==1)then

if (T>T2)then

ydot(36)=0;

ydot(37)=0;

end if

end if

ydot(38) = -J_serca*Vsr/Vmyo-J_CaB_cytosol +J_ca_slmyo/Vmyo*(ypre(37)-ypre(38)); ! [mM/msec]

!! Membrane Potential

I_Na_tot = I_Na_tot_junc + I_Na_tot_sl; ! [uA/uF]

I_Cl_tot = I_ClCa+I_Clbk; ! [uA/uF]

I_Ca_tot = I_Ca_tot_junc+I_Ca_tot_sl;

I_tot = I_Na_tot+I_Cl_tot+I_Ca_tot+I_K_tot;

ydot(39) = -(I_tot+I_app());

if(V_manipulate==1)then

if (T>=T2)then

ydot(39)=0;

end if

end if

end subroutine

function I_app()

use para

implicit none

real*8::I_app

integer::n

if(T>=0.and.T<1)then

I_app=-40

else

I_app=0

end if

return

end
