# Supplementary material for: Dissecting the roles of calcium cycling and its coupling with voltage in the genesis of early afterdepolarizations in cardiac myocyte models
Source: PLoS Comput Biol. 2024 Feb 28;20(2):e1011930. doi: 10.1371/journal.pcbi.1011930 (PMC10927084; doi:10.1371/journal.pcbi.1011930)
Supplement: Attachment — Submitted filename: response.pdf [file pcbi.1011930.s003.pdf]

We thank the editor for inviting us to resubmit a revision of our manuscript for consideration for publication and the reviewers for their constructive comments. We have revised our manuscript following the suggestions of the reviewers as detailed by the point-to-point response below. We also added our computer code of the model as a supplement.

Response to Reviewer #1:

In the article entitled "Dissecting the roles of calcium cycling and its coupling with voltage in the genesis of early afterdepolarizations in cardiac myocyte model" by Wang et al, the authors categorize four different mechanisms for the development of EAD. The reviewer believes that this paper is well written; however, this paper contains problems with methodological rigor, and the reviewer has concerns about the results and its conclusions. Therefore, I do not think they are suitable for publication as is in PLoS Computational Biology.

We thank the reviewer for the positive comments. We have improved our manuscript following the reviewer's suggestions.

The main concern of this manuscript is that the methodology adopted in the present study includes a certain kind of problem. It is a well-known fact that initial conditions (initial value sets of state variables) are extremely important in simulations (numerical integrations) in ordinary differential equation models. Depending on the parameter set, it is not surprising to observe bistable or tri-stable dynamics depending on their initial values (e.g. Tsumoto et al., Sci Reps, 2017). The results presented in this manuscript shown here have been obtained by a huge number of simulations though, no data are provided regarding the initial values at which the simulations were performed. The authors should provide initial values (initial conditions) for each simulation.

We used a single set of initial conditions for all simulations for each model. The initial values are added in Supplemental Information for each model.

As a question, were these simulations performed with identical initial values (initial conditions) for each simulation? If so, how significant is it to classify the transient dynamics obtained by single stimulation under identical initial conditions? For example, could changing the initial conditions change dynamics classified as Vm-driven mechanisms to dynamics due to other EAD mechanisms? To dispel these doubts, authors should classify the mechanism of EAD development when the action potential response of cardiomyocyte models reaches a steady state after repeated application of multiple stimuli. The reviewer believes that the mechanisms of EAD at steady-state would be most reliable if they could be explained individually.

We thank the reviewer for raising this important question. As stated in our response to the comment above, all simulations were performed by using the same set of initial values. These initial values are the same as those used in the original paper of the WG model by Wilson et al. As pointed out by the reviewer, the EAD behaviors/mechanisms can be initial condition dependent. To show how initial conditions (or pacing protocols) affect EAD behaviors, we carried out simulations using two other pacing protocols, i.e., the so-called S1S1 and S1S2 pacing protocols. We used these two protocols to redo the simulations shown in Fig.2 and presented the new results as a new Fig.3. As shown in Fig.3, under these two pacing protocols, the phase

diagrams are similar as that shown in Fig.2, i.e., the same EAD mechanisms are present in the phase diagrams but the locations (or parameters) for different EAD mechanisms are different. In other words, although the EAD behaviors/mechanisms depend on initial conditions or pacing protocols (as well as the S1S1 interval and S1S2 interval), the same EAD mechanisms remain regardless of the pacing protocol used. However, under either of the pacing protocols, complex behaviors (such as n:m responses, alternans and chaos) can occur during the S1 pacing period, which makes it difficult to uniquely define the phase diagram. This was one of the reasons that we used a single set of initial condition for all simulations. We also discussed the initial condition/pacing protocol issue in Discussion (lines 306-314).

Minor.

1. The reviewer could not understand the schematic in Fig.1A. What does the outermost line mean? (Does it mean the cell membrane?) If so, what space does the SL space between the myoplasm and the plasma membrane mean?

The outermost boundary is the cell membrane. In the WG (and the previous SB) model, the submembrane space is divided into two compartments, i.e., the JXN space and the SL space. JXN is the cleft adjacent to the junctional SR, and SL is the remaining submembrane space. Ca^{2+} diffuses very fast between the two spaces, and for simplicity, we just treat them as a single space, and thus $[Ca]_{JXN}=[Ca]_{SL}=[Ca]_{sub}$. We revised Fig.1A and the caption for better clarification.

2. Does JXN space mean the subspace in the cytoplasm where the t-tubule membrane and SR membrane (junctional SR membrane) are close together?

Yes. Please see the revised Fig.1 caption.

3. Does JCaslmyo mean Ca influx into the cell via L-type Ca channels?

No. JCaslmyo means the Ca flux from SL space to cytoplasm via diffusion. To avoid confusion, we redraw Fig.1A and explain its meaning in line 75.

4. To put a finer point on it, the reviewer can imagine that ‘ctrl’ means ‘control’, but it should be defined properly.

We revised it by using the full term, please see lines 74-79.

5. The meaning of the lines in the lower panel in Fig. 2C are reversed: the blue line is the highest and the red line is the lowest level.

Thanks for the reviewer’s careful reading. We corrected this error.

6. In each phase diagram, what dynamics occur in the gray region? Please add an explanation.

The grey region means repolarization failure (RF). For better clarification, we added schematic APs in Fig.2A. However, in the phase diagrams in Fig.3, we define RF as APD>S1S1 interval.

Response to Reviewer #2:

In this study by Wang and colleagues, the authors investigate early afterdepolarizations (EADs) in cardiac myocyte models, considering different parameter regimes and highlighting 4 different mechanisms underlying EADs.

The study demonstrates both previously identified and new mechanisms for EAD generation, the respective parameter conditions needed for each mechanism, and also identifies the presence (or absence) of these mechanisms in several different well-established cardiac models.

The study is rigorous and the manuscript well written. My comments are primarily focused on data presentation and clarification of methods.
We thank the reviewer for the positive comments. We have improved our manuscript following the reviewer's suggestions.

1. In Figure 2A, please clarify the model dynamics in the grey and white regions of the phase diagram (either in the text or figure caption). Presumably, these are repolarization failure and normal APs, but please clarify.
The grey region means repolarization failure (RF) and the white region means normal repolarization. For better clarification, we added schematic APs in Fig.2A. However, in the phase diagrams in Fig.3, we define RF as APD>S1S1 interval.

2. Methods: Was some automated analysis used to identify the mechanisms in the different phase diagrams and the Monte Carlo study (of other models), or were all simulations analyzed individually? Please clarify.
If automated, please describe the specific criteria used for classification in the Methods.
All simulations were analyzed individually. The statement is added in the end of the "Models and methods" section (lines 90-95).

3. Figure 3B: Can the authors add another vertical line near the start of the oscillations? It is not obvious that the initial increase in Ca_sub leads the initial increase in Vm, which is a key aspect of this mechanism.
Done, appearing in the revised Fig.4B, where $[Ca]_{sub}$ slightly precedes V_m in the initial oscillation.

4. Table 1: Please clarify how many simulations were performed for each model using the Monte Carlo approach.
Done.

5. As a general comment, the Discussion and Conclusions section is quite brief. I appreciate the succinct summary of the 4 mechanisms, but I think it would be valuable for the authors to provide some additional physiological context to the study, in particular related to the feasibility of the different EAD mechanisms under different pathological conditions.
We added a paragraph in Discussion to address the reviewer's point (lines 315-332).

Minor: In Figure 2C, please confirm the 'lowest' and 'highest' labels in the caption. They appear to be switched.
Thanks for the careful reading. The mistake is corrected.

Minor: Figure 3E: 'clamped' is misspelled in the figure titles

Corrected.
